# Supplementary material for: Parent-infant observation for prediction of later childhood psychopathology in community-based samples: A systematic review
Source: PLoS One. 2022 Dec 29;17(12):e0279559. doi: 10.1371/journal.pone.0279559 (PMC9799315; doi:10.1371/journal.pone.0279559)
Supplement: S2 File — (DOCX) [file pone.0279559.s002.docx]

| Search ID | Search |
| --- | --- |
| 1 | MeSH descriptor: [Cohort Studies] explode all trees |
| 2 | MeSH descriptor: [Psychopathology] explode all trees |
| 3 | MeSH descriptor: [Mental Disorders] explode all trees |
| 4 | MeSH descriptor: [Infant] explode all trees |
| 5 | MeSH descriptor: [Psychiatry] explode all trees |
| 6 | (cohort* or "birth cohort*").ti,ab |
| 7 | (mental* or psychiatr* or psychopath* or conduct*).ti,ab |
| 8 | (baby or babies or infant* or infancy or newborn*).ti,ab |
| 9 | #1 or #6 |
| 10 | #2 or #3 or #5 or #7 |
| 11 | #4 or #8 |
| 12 | #9 and #10 and #11 |
| 13 | (marker* or "risk factor*" or predict* or associat* or precursor* or antecedent* or link*).ti,ab |
| 14 | #12 and #13 |

**Full electronic search strategy**

**Cochrane Library**: yielded 3335 articles

**EMBASE**: yielded 5223 articles

| Search ID | Search |
| --- | --- |
| 1 | exp cohort analysis/ |
| 2 | exp mental disease/ |
| 3 | exp psychiatry/ |
| 4 | exp psychiatric diagnosis/ |
| 5 | exp "Diagnostic and Statistical Manual of Mental Disorders"/ |
| 6 | exp infant/ |
| 7 | exp newborn/ or exp newborn period/ |
| 8 | exp baby/ |
| 9 | (infancy or infant* or baby or babies or newborn*).ti,ab. |
| 10 | (later adj3 (mental*or psychopath* or psychiatr* or conduct*)).ti,ab. |
| 11 | ("birth cohort*" or cohort*).ti,ab. |
| 12 | 1 or 11 |
| 13 | 2 or 3 or 4 or 5 or 10 |
| 14 | 6 or 7 or 8 or 9 |
| 15 | 12 and 13 and 14 |
| 16 | (marker* or "risk factor*" or predict* or parameter* or associat* or antecedent* or link*).ti,ab. |
| 17 | 15 and 16 |

**CINAHL**: yielded 3768 articles

| Search ID | Search |
| --- | --- |
| 19 | (MH "Mental Disorders Diagnosed in Childhood+") |
| 18 | S14 AND S15 AND S16 AND S17 |
| 17 | S12 OR S13 |
| 16 | S1 OR S10 OR S11 |
| 15 | S19 OR S2 OR S8 OR S9 |
| 14 | S3 OR S4 OR S5 OR S6 OR S7 |
| 13 | AB marker* or "risk factor*" or link* or associat* or antecedent* or precursor* |
| 12 | TI marker* or "risk factor*" or link* or associat* or antecedent* or precursor* |
| 11 | AB baby or babies or infant* or infancy or newborn* |
| 10 | TI baby or babies or infant* or infancy or newborn* |
| 9 | AB mental* or psychiatr* or psychopath* or conduct* |
| 8 | TI mental* or psychiatr* or psychopath* or conduct* |
| 7 | TI "birth cohort*" |
| 6 | TI cohort* |
| 5 | AB "birth cohort*" |
| 4 | AB cohort* |
| 3 | (MH "Prospective Studies+") |
| 2 | (MH "Psychopathology") |
| 1 | (MH "Infant+") |

**PsycINFO**: yielded 1145

| Search ID | Search |
| --- | --- |
| 1 | exp Cohort Analysis/ |
| 2 | (cohort* or "birth cohort*").ti,ab. |
| 3 | exp Mental Disorders/ |
| 4 | exp "Diagnostic and Statistical Manual"/ |
| 5 | exp Psychopathology/ |
| 6 | exp Psychiatric Symptoms/ |
| 7 | (mental* or psychiatr* or psychopath* or psychiatr* or conduct*).ti,ab. |
| 8 | 1 or 2 |
| 9 | 3 or 4 or 5 or 6 or 7 |
| 10 | (baby or babies or infant* or infancy or newborn*).ti,ab. |
| 11 | ("risk factor*" or predict* or associat* or link* or precursor* or antecedent* or marker*).ti,ab. |
| 12 | 8 and 9 and 10 and 11 |

**MIDIRS**: yielded 1693 articles

| Search ID | Search |
| --- | --- |
| 1 | Cohort studies.de. |
| 2 | ("birth cohort*" or cohort*).ti,ab. |
| 3 | Mental disorders.de. |
| 4 | Psychopathology.de. |
| 5 | (mental* or psychiatr* or psychopath* or conduct*).ti,ab. |
| 6 | Infant.de. |
| 7 | newborn.de. |
| 8 | Infant - newborn.de. |
| 9 | (infant* or infancy or baby or babies or newborn*).ti,ab. |
| 10 | (marker* or "risk factor*" or predict* or associat* or link* or antecedent* or precursor*).ti,ab. |
| 11 | 1 or 2 |
| 12 | 3 or 4 or 5 |
| 13 | 6 or 7 or 8 or 9 |
| 14 | 10 and 11 and 12 and 13 |

**MEDLINE**: yielded 8068 articles

| Search ID | Search |
| --- | --- |
| 1 | exp Cohort Studies/ |
| 2 | (cohort* or "birth cohort*").ti,ab. |
| 3 | exp Mental Disorders/ |
| 4 | exp Psychiatry/ |
| 5 | exp Psychopathology/ |
| 6 | (later adj3 (mental* or psychiatr* or psychopath* or conduct*)).ti,ab. |
| 7 | exp Infant, Newborn/ or exp Infant/ |
| 8 | (baby or babies or infant* or infancy or newborn*).ti,ab. |
| 9 | 1 or 2 |
| 10 | 3 or 4 or 5 or 6 |
| 11 | 7 or 8 |
| 12 | (marker* or precursor* or predict* or associat* or link* or "risk factor*" or antecedent*).ti,ab. |
| 13 | 9 and 10 and 11 and 12 |
