# Supplementary material for: Parent-infant observation for prediction of later childhood psychopathology in community-based samples: A systematic review
Source: PLoS One. 2022 Dec 29;17(12):e0279559. doi: 10.1371/journal.pone.0279559 (PMC9799315; doi:10.1371/journal.pone.0279559)
Supplement: S3 File — (RTF) [file pone.0279559.s003.rtf]

S I G N	Methodology Checklist 3: Cohort studies	
Study identification  (Include author, title, year of publication, journal title, pages)
	
Data extraction form for: 
Systematic review of ability of assessments of parent-child interaction in the first year of life to predict subsequent childhood psychopathology	
Checklist completed by: 	
BASIC CRITERIA (new section for P-C Interaction review)	
Study Characteristics	Eligibility Criteria	Criteria Met? (Y, N, Unclear)	Location in source	
Type of Study	Prospective birth or population-based cohort 

Exclude: 
Disease cohort
High risk cohort
Retrospective studies			
Participants	Infants in first year of life

Exclude:
Pre-term, SGA or LWB cohort
Deprived/institutionalised background
Cohort with mentally unwell parents			
Type of assessment	Independent assessment of parent-child interaction during first year of life

Exclude:
Questionnaire-based or parent-rated assessments 			
Outcome	Assessment of child psychopathology, psychiatric symptoms or behaviour between ages 1-18			
Include or Exclude (please provide reason):	
IF EXCLUDING THEN PROCEED NO FURTHER	
	
Section 1:  Internal validity	
In a well conducted cohort study:	Does this study do it?	
1.1	The study addresses an appropriate and clearly focused question.	Yes  □
Can't say □	No □
	
Selection of subjects	
1.2	The participants being studied are representative of the general population they have been taken from.	Yes  □
Can't say □	No □
Does not apply □	
1.3	The study indicates how many of the people asked to take part did so.
	Yes  □
	No □
Does not apply □	
1.4	The likelihood that some eligible subjects might have the outcome at the time of enrolment is assessed and taken into account in the analysis.	Yes  □
Can't say □	No □
Does not apply □	
1.5	What percentage of individuals dropped out before the study was completed.		
1.6	Comparison is made between full participants and those lost to follow up.	Yes  □
Can't say □	No □
Does not apply □	

ASSESSMENT	
1.7	The outcomes are clearly defined.	Yes  □
Can't say □	No □
	
1.8	The assessment of outcome is made blind to initial assessment status. 	Yes  □
Can't say □	No □
Does not apply □	
1.9	Where blinding was not possible, there is some recognition that knowledge of exposure status could have influenced the assessment of outcome.	Yes  □
Can't say □	No □
□	
1.10	The methods of assessments are reliable.	Yes  □
Can't say □	No □
	
1.11	Evidence from other sources is used to demonstrate that the method of outcome assessment is valid and reliable.	Yes  □
Can't say □	No □
Does not apply□	
CONFOUNDING	
1.13	The main potential confounders are identified and taken into account in the design and analysis.	Yes  □
Can't say □	No □
	
STATISTICAL ANALYSIS	
1.14	Have confidence intervals been provided?	Yes  □	No □	
Section 2:  OVERALL ASSESSMENT OF THE STUDY	
2.1	How well was the study done to minimise the risk of bias or confounding? Rate the overall methodological quality of the study, using the following as a guide: High quality (++): Majority of criteria met. Little or no risk of bias.  Results unlikely to be changed by further research. Acceptable (+): Most criteria met. Some flaws in the study with an associated risk of bias, Conclusions may change in the light of further studies. Low quality  (0): Either most criteria not met, or significant flaws relating to key aspects of study design. Conclusions likely to change in the light of further studies.
	High quality (++) □
Acceptable (+) □
Unacceptable – reject 0 	
2.2	Taking into account clinical considerations, your evaluation of the methodology used and the statistical power of the study, do you think there is clear evidence of an association between the results of the initial assessment and the outcome?	Yes           No 
Can't say 	
SECTION 3: DESCRIPTION OF THE STUDY	
	Description
Include comparative information for each intervention or comparison group if available	Location in text or source (pg & ¶/fig/table/other)	
Population description
(from which study participants are drawn)	     	     	
Setting
(including location and social context)	     	     	
Inclusion criteria 	     	     	
Exclusion criteria	     	     	
Method of recruitment of participants 	     	     	
Informed consent obtained			
Yes	No	Unclear
     	     	
Total participants at start of study 	     	     	
Sex	     	     	
Race/Ethnicity	     	     	
Other relevant sociodemographics	     	     	
Notes:        

	
SECTION 4: DESCRIPTION OF ASSESSMENT	
At what age are infants assessed?		
What is assessed?		
Who is assessing them?		
What measure is used to assess them? Please provide details including if it is validated.		
What were the results?		
SECTION 5: DESCRIPTION OF OUTCOME	
At what age are children assessed?		
What is assessed?		
Who is assessing them?		
What measure is used to assess them? Please provide details including if it is validated.		
What are the results?		
How long were children followed up for? Was this sufficie nt?		
How many were lost to follow up and what was done with this missing data?		
Describe any statistical analysis done including the appropriateness.		
Any other results reported?		
SECTION 6: FINAL POINTS	
What were the authors' conclusions?		
Study funding sources.		
Conflicts of interests		
Notes: 	
