# Supplementary material for: Parent-infant observation for prediction of later childhood psychopathology in community-based samples: A systematic review
Source: PLoS One. 2022 Dec 29;17(12):e0279559. doi: 10.1371/journal.pone.0279559 (PMC9799315; doi:10.1371/journal.pone.0279559)
Supplement: S4 File — (DOCX) [file pone.0279559.s004.docx]

**Association of variables with later childhood psychopathology**

| **Study** | **Variable** | **Statistical Test** | **Result** |
| --- | --- | --- | --- |
| 1 | Parent-infant interaction:  1) positive interaction  2) negative interaction | Odds ratio (95% confidence intervals) | **1) 0.85 (0.74-0.96)**  2) 0.98 (0.60-1.51) |
| 2 | Likelihood of being a ‘case’ | Fisher’s exact test | p = 0.238 |
| 3 | Maternal-infant interaction | Odds ratio (95% confidence intervals) | 1.91 (0.97-3.77) |
| 5 | Parent-infant interaction:  1) Child vocalisation frequency  2) Parent vocalisation frequency | Odds ratio (95% confidence intervals) | 1) 1.38 (0.88-2.19)  **2) 0.69 (0.52-0.90)** |
| 6 | Parent-infant joint attention:  1) shared look rate  2) shared attention rate  3) shared attention intensity | Odds ratio (95% confidence intervals) | 1) 1.2 (0.9-1.7)  2) 1.0 (0.7-1.5)  3) 0.8 (0.6-1.2) |
| 7 | Maternal-infant interaction:  1) adult and infant cooperativeness  2) adult speech and activity | Odds ratio (95% confidence intervals) | 1) 0.84 (0.61-1.17)  **2) 0.57 (0.40-0.80)** |

**Association of variables with childhood emotional disorders**

| **Study** | **Variable** | **Statistical test** | **Result** |
| --- | --- | --- | --- |
| 1 | Positive parent-infant interaction | Odds ratio (95% confidence intervals) | **0.82 (0.66-0.98)** |
| 2 | Likelihood of being a ‘case’ | Fisher’s exact test | p = 1.000 |
| 3 | Maternal-infant interaction | Odds ratio (95% confidence intervals) | 1.67 (0.63-4.44) |
| 5 | Parent-infant interaction:  1) Child vocalisation frequency  2) Parent vocalisation frequency | Odds ratio (95% confidence intervals) | 1) 0.89 (0.42-1.71)  **2) 0.63 (0.42-0.92)** |
| 6 | Parent-infant joint attention:  1) shared look rate  2) shared attention rate  3) shared attention intensity | Odds ratio (95% confidence intervals) | 1) 1.3 (0.8-1.9)  2) 1.2 (0.7-2.2)  3) 0.9 (0.5-1.7) |
| 7 | Maternal-infant interaction:  1) adult and infant cooperativeness  2) adult speech and activity | Odds ratio (95% confidence intervals) | 1) 1.15 (0.70-1.98)  **2) 0.51 (0.31-0.80)**  Anxiety disorders:  1) 1.13 (0.67-1.97)  **2) 0.47 (0.28-0.75)** |
| 9 | 1) Correlation between Maternal sensitivity and later child separation anxiety  2) Prediction of separation anxiety from maternal sensitivity | 1) Pearson product moment correlation coefficient  2) Regression analysis | **1)** **-0.29 (p <0.01)**  2) B -0.06, SE 0.09, β -0.07 (p>0.1) |

**Association of variables with later disruptive behaviour disorders**

| **Study** | **Variable** | **Statistical test** | **Result** |
| --- | --- | --- | --- |
| 1 | Positive parent-infant interaction | Odds ratio (95% confidence intervals) | **0.84 (0.71-0.97)** |
| 2 | Likelihood of being a ‘case’ | Fisher’s exact test | p = 0.069 |
| 3 | Maternal-infant interaction | Odds ratio (95% confidence intervals) | 1.63 (0.52-5.12) |
| 5 | Parent-infant interaction:  1) Child vocalisation frequency  2) Parent vocalisation frequency | Odds ratio (95% confidence intervals) | **1) 1.77 (1.07-3.05)**  **2) 0.68 (0.47-0.94)** |
| 6 | Parent-infant joint attention:  1) shared look rate  2) shared attention rate  3) shared attention intensity | Odds ratio (95% confidence intervals) | 1) 1.3 (0.9-1.9)  2) 1.0 (0.7-1.6)  3) 0.7 (0.5-1.1) |
| 7 | Maternal-infant interaction:  1) adult and infant cooperativeness  2) adult speech and activity | Odds ratio (95% confidence intervals) | 1) 0.75 (0.52-1.09)  **2) 0.53 (0.34-0.79)** |

**Association of variables with oppositional defiant and conduct disorders**

| **Study** | **Variable** | **Statistical test** | **Result** |
| --- | --- | --- | --- |
| 1 | Positive parent-infant interaction | Odds ratio (95% confidence intervals) | **0.81 (0.65-0.97)** |
| 2 | Likelihood of being a ‘case’ | Fisher’s exact test | p = 0.169 |
| 5 | Parent-infant interaction:  1) Child vocalisation frequency  2) Parent vocalisation frequency | Odds ratio (95% confidence intervals) | 1) 1.69 (0.97-3.02)  **2) 0.64 (0.41-0.94)** |
| 6 | Parent-infant joint attention:  1) shared look rate  2) shared attention rate  3) shared attention intensity | Odds ratio (95% confidence intervals) | **1) 1.5 (1.0-2.3)**  2) 1.1 (0.7-1.8)  3) 0.8 (0.5-1.2) |
| 7 | Maternal-infant interaction:  1) adult and infant cooperativeness  2) adult speech and activity | Odds ratio (95% confidence intervals) | 1) 0.76 (0.50-1.17)  **2) 0.50 (0.31-0.80)**  Conduct disorder:  1) 0.70 (0.32-1.56)  **2) 0.37 (0.12-0.98)** |
| 8 | Maternal responsiveness | Longitudinal Poisson regression models with GEE methodology | β -0.14, z -1.34, p = 0.18 |

**Association of variables with attention deficit hyperactivity disorders (ADHD) and hyperkinetic disorders**

| **Paper** | **Variable** | **Statistical test** | **Result** |
| --- | --- | --- | --- |
| 2 | Likelihood of being a ‘case’ | Fisher’s exact test | 1) any ADHD p = 0.578  **2) Inattentive ADHD p = 0.039** |
| 3 | Maternal-infant interaction | Odds ratio (95% confidence intervals) | **5.20 (1.55-17.47)** |
| 5 | Parent-infant interaction:  1) Child vocalisation frequency  2) Parent vocalisation frequency | Odds ratio (95% confidence intervals) | 1) 1.76 (0.93-3.41)  2) 0.69 (0.43-1.06) |
| 6 | Parent-infant joint attention:  1) shared look rate  2) shared attention rate  3) shared attention intensity | Odds ratio (95% confidence intervals) | 1) 1.1 (0.6-1.7)  2) 0.9 (0.5-1.6)  3) 0.6 (0.4-1.1) |
| 7 | Maternal-infant interaction:  1) adult and infant cooperativeness  2) adult speech and activity | Odds ratio (95% confidence intervals) | 1) 0.88 (0.52-1.57)  **2) 0.42 (0.23-0.70)**  Inattentive ADHD:  1) 0.68 (0.34-1.43)  **2) 0.19 (0.04-0.56)** |

**Association of variables with pervasive developmental disorders**

| **Study** | **Variable** | **Statistical test** | **Result** |
| --- | --- | --- | --- |
| 2 | Likelihood of being a ‘case’ | Fisher’s exact test | p = 1.000 |
| 3 | Maternal-infant interaction | Odds ratio (95% confidence intervals) | 2.90 (0.97-8.68) |
| 5 | Parent-infant interaction:  1) Child vocalisation frequency  2) Parent vocalisation frequency | Odds ratio (95% confidence intervals) | 1) 1.30 (0.47-3.12)  2) 0.76 (0.38-1.36) |
| 6 | Parent-infant joint attention:  1) shared look rate  2) shared attention rate  3) shared attention intensity | Odds ratio (95% confidence intervals) | 1) 1.2 (0.4-2.0)  2) 0.6 (0.2-1.6)  3) 1.6 0.5-6.9) |
| 7 | Maternal-infant interaction:  1) adult and infant cooperativeness  2) adult speech and activity | Odds ratio (95% confidence intervals) | 1) 1.23 (0.54-3.58)  2) 0.56 (0.24-1.43) |
| 4 | 1) PREAUT 4 to predict Autistic Spectrum Disorders  2) PREAUT 9 to predict Autistic Spectrum Disorders  3) PREAUT 4 and Pervasive developmental disorders  4) PREAUT 9 and Pervasive developmental disorders | a) Positive predictive value (%)  b) Sensitivity  c) Specificity | 1a) 25.4-26.3  1b) 16.0-20.6  1c) 99.6-99.6  2a) 20.2-36.4  2b) 30.5-41.2  2c) 99.3-99.4  3a) 51.8-52.6  3b) 23.5-28.4  3c) 99.8-99.8  4a) 33.12-45.4  4b) 33.8-37.9  4c) 99.4-99.5 |
