# Supplementary material for: Parent-infant observation for prediction of later childhood psychopathology in community-based samples: A systematic review
Source: PLoS One. 2022 Dec 29;17(12):e0279559. doi: 10.1371/journal.pone.0279559 (PMC9799315; doi:10.1371/journal.pone.0279559)
Supplement: Attachment — Submitted filename: Response to Reviewers.docx [file pone.0279559.s005.docx]

Response to Reviewers

| **Reviewers comment:** | **Response:** |
| --- | --- |
| 1. The title, Abstract, and Introduction do not clearly explain what 'low-risk' populations are. I think that the authors should make this clear throughout. | Line 3 (Title)  Revisions made:  Parent-infant observation for prediction of later childhood psychopathology in community-based samples: A Systematic Review  Line 29 (Abstract)  Revisions made:  ‘Included studies were those recruiting from population or community-based birth cohort data, which we define as ‘low-risk’. Studies based on populations known to have a diagnosis of psychiatric illness or developmental disorder, or at high genetic or environmental risk of being diagnosed with such disorder, were excluded.’  Line 105 (Introduction)  Revisions made:  ‘Here, we define ‘low-risk’ samples as those recruited from population or community-based birth cohort data and not from populations known to have a diagnosis of psychiatric illness or developmental disorder, or at high genetic or environmental risk of these disorders.’ |
| 2. I would expect to see the authors provide clear information regarding PECO in the Methods section. | We do not consider that the standard PECO approach applies easily to our study design since the included studies either use case-control or retrospective cohort designs. There are therefore no comparator groups in terms of differential exposure. We have nevertheless tried to clarify in the text and in Table 1, for each included study, the Population, the Exposures included in the analysis, and the Outcomes under study. |
| 3. I am not sure why the authors want to mention ACEs in the Introduction. Apparently, ACEs are not the focus of the present systematic review, nor did the authors discuss the ACEs among parent-child interaction. | The Introduction has been edited to remain relevant to the scope of this review. Discussion of ACEs has been removed and the focus redirected onto insecure attachment and observable problems in parent-infant interaction.  Line 47 (Introduction)  Revisions made:  Insecure parent-child attachment is associated with higher disease burden in adulthood [1, 2]. As such, problems in parent-child interaction can act as a potential early observable risk factor, and target for early intervention [3]. Research has shown that parenting interventions have been found to be an effective method of improving parent-child interactions, emotional and behavioural adjustment of children and the psychosocial well-being of parents [4]. |
| 4. The authors did not mention who screened the title/abstract and was this done by different reviewers independently? | Line 190 (Methods)  Revisions made: ‘One reviewer designed the search strategy and screened the results based on reading the titles and abstracts. The same reviewer then assessed the papers identified for full screening. Two other reviewers independently assessed half of the full text papers each for eligibility, whilst blind to the other reviewer’s decision. A third reviewer arbitrated on any disagreements.’ |
| 5. The authors can elaborate more on the risk of bias assessment tools used for this study. Where does it come from and did they make adaptations? | We stated that the data extraction form incorporated a risk of bias assessment adapted from the SIGN checklist. RevMan software from the Cochrane Collaboration was used to display the results of this assessment graphically. We have amended the manuscript to clarify that we simply edited the SIGN checklist to exclude items that were not relevant to our study design. Our data extraction form is included as an appendix. |
| 6. It is unfortunate that these authors were not able to conduct quantitative synthesis owing to the limited numbers of studies included. The authors may discuss the appropriateness of inclusion/exclusion defined for this study. Why excluding parent-reported measures? Why excluding high risk samples? I understand that this cannot be changed at the current stage. Would a review with broader inclusion criteria needed in the future to examine this research question? And then the authors can carefully examine each subgroup within such a review. | “Why excluding parent-reported measures?”  We have clarified the aim of the review throughout the paper:  Line 22 (Abstract)  Revisions made: ‘This review aimed to assess the utility of current observational methods used to assess parent-child interactions – within the first year of life – and their ability to screen and identify children from low-risk samples most at risk of developing childhood psychopathology.  Line 65 (Introduction)  Revisions made: ‘Observational measures are also particularly sensitive to changes in parent-infant interaction over time, much greater than those detected by parent self-reported measures [9].’  Line 157 (Methods)  Revisions made: ‘To have most relevance to primary care clinicians, we limited the scope of this review to papers that assessed parent-child interaction through direct observation. This could include any measure of parent-infant interaction, such as level of maternal and infant speech, gaze or activity level and was not restricted to measures of parental sensitivity. As discussed previously, parent self-report measures are widely known to involve a high degree of bias and can be greatly impacted by the parent’s mood at the time of assessment, so observations made by a neutral observer may have particular value. [9, 10] This review has particular relevance to those working in primary care, where direct observation of parent-child interactions throughout routine care appointments is possible.  “Why excluding high risk samples?”  Line 139 (Methods):  ‘As such, we have identified a gap within the current literature and wish to extend the research on the association between parent-infant interaction and later childhood psychopathology to include those from ‘low-risk’ populations. Positive and negative predictive values of observations vary markedly with prevalence and are thus very different in high-risk and whole population samples. Therefore, data from ‘high-risk’ studies are likely to have limited value for primary care clinicians dealing mainly with unaffected individuals. Accordingly, we will examine population or birth cohort data that include a range of psychiatric diagnoses as an outcome measure and have excluded papers focusing on ‘high-risk’ groups.’ |
